# Firework injuries around New Year’s Eve - epidemiology, injury patterns and risk factors

**DOI:** 10.1007/s00068-025-02785-y

**Published:** 2025-02-13

**Authors:** Helena Wegmann, Steffi Mayer, Notker Blankenburg, Peter Zimmermann, Torsten Schulz, Martin Lacher, Christian Kleber, Georg Osterhoff

**Affiliations:** 1 Department of Orthopaedics, Trauma and Plastic Surgery, University Hospital Leipzig, 04103 Leipzig, Germany; 2 Department of Pediatric Surgery, University Hospital Leipzig, Leipzig, Germany

**Keywords:** Fireworks, Wounds and injuries, Burns, Emergency service, Epidemiology, Open fractures

## Abstract

**Introduction:**

This study aims to determine key demographic and behavioural risk factors contributing to firework-related injuries and their outcomes.

**Methods:**

A retrospective study was conducted on pediatric and adult patients treated for firework-related injuries at the University Hospital Leipzig from December 28th to January 3rd over 10 years (2013–2023). Data were collected on demographics, injury details, weather conditions and classification of fireworks.

**Results:**

A total of 155 patients (mean age 24 years, 80% male) were included. Injuries predominantly occurred within two hours after midnight, and all incidents involved violation of safety regulations. Most injuries were self-inflicted (48.4%), with hands being the most frequently affected body part (52.9%). Burn injuries were common (43.9%) but were less likely during rainy weather (OR 0.4, 95% CI 0.2–0.7, *p* = 0.004). Surgical intervention was required in 23.9% of cases, with significant risk factors being associated bone injuries (OR 107.1, 95% CI 22.7 to 505.6), male sex (OR 5.7, 95% CI 1.3–25.0) and multiple body region injuries (OR 4.9, 95% CI 2.1–11.7). Permanent loss of function was observed in 46.5% of all patients.

**Conclusion:**

Firework-related injuries around New Year’s Eve are associated with significant morbidity, particularly affecting the hands and often requiring surgical intervention. Bone injuries, male sex and multiple body region injuries were associated with higher need for surgical treatment. Despite most fireworks being purchased legally, there is widespread disregard to age restrictions and safety protocols. These findings highlight the necessity of public health initiatives to reduce the incidence and severity of such injuries, rather than stricter regulations.

## Introduction

Firework-related injuries represent a significant public health concern during the New Year’s Eve period, a time traditionally associated with celebratory use of fireworks. In Germany, fireworks are primarily used during and around the celebrations held on New Year’s Eve. The timing of sales and the permitted use of fireworks on December 31st and January 1st aligns with this cultural practice. This period marks a substantial increase in the incidence and severity of such injuries, leading to considerable morbidity and workload of emergency departments that have significant implications for public health. Most firework-related injuries detected in our study were caused by consumer fireworks/category F2 (e.g. rockets, fountains). These fireworks can only be purchased legally during the specified time frame (December 28th– December 31st ) in Germany and their use is restricted to December 31st and January 1st [8]. The establishment of a limited timeframe for the sale and use of fireworks by regulatory authorities is intended to mitigate the potential risks associated with these pyrotechnics while maintaining the celebratory traditions.

Previous studies identified hands and eyes as the most affected areas of the body in firework-related injuries, frequently resulting in severe trauma such as loss of hand function and blindness [1, 2]. Ears have been found to be the second most affected body part [3–7]. Despite the well-documented risks associated with fireworks, they remain widely used in Germany [8], and their import has even increased significantly for New Year’s Eve purposes [9]. The use of fireworks is often associated with inadequate safety precautions, especially regarding non-compliance with legal restrictions such as age restrictions and the observance of safety distances [10] or the handling of fireworks under the influence of drugs or alcohol [11, 12].

The debate around the over-the-counter availability of fireworks has intensified in recent years, as well as the amount of freely available fireworks purchased in Germany after the COVID-19 pandemic [10, 13, 14]. There are growing safety concerns particularly in densely populated urban areas where improper use can lead to serious injuries not only for users but also for bystanders [1, 15]. These concerns have led to discussions about stricter regulations or even outright bans on the sale and use of consumer fireworks, especially considering the COVID-19-related restrictions of the latter which have significantly reduced trauma injuries to the eye in Germany [16]. Understanding the epidemiology of firework-related injuries, identifying common injury patterns, and pinpointing key risk factors is crucial for shaping public health policies and regulatory measures.

In summary, this study seeks to fill critical gaps in the existing literature by offering a comprehensive analysis of firework-related injuries during New Year’s celebrations, with the aim of informing public health strategies and contributing to the development of more effective safety regulations.

## Materials and methods

### Patients

The protocol of this study was approved by the local ethics committee (reference 084/23-ek).

Consecutive pediatric and adult patients (a total of around 7300 patients) who were seen by the pediatric surgeons or trauma surgeons in the emergency departments of the University Hospital Leipzig between December 28th and January 3rd (the time around New Year’s Eve extended to a total of seven days to include all instances of premature ignition or ignition subsequent to New Year’s Eve) during the period from 2013 to 2023 for a fireworks injury were retrospectively screened manually using the clinical information system of University Hospital in Leipzig. Patients with injuries documented and submitted as “burn injuries”, “polytrauma” or “fracture” were flagged and identified, and their files were subjected to further evaluation. Patients with isolated firework-related injuries to the eyes or hearing damage were not included in the analysis.

In addition, patients who had objected to the use of their personal data were excluded.

### Data acquisition

The following details were analyzed: patient specifics and demographic characteristics, including the day of injury and corresponding weather data (German weather service location Leipzig-Holzhausen, Germany [17, 18]); location and severity of injury, identified with the use of a standardized classification system (Abbreviated Injury Scale [19]); cause and mechanism of fireworks injury (e.g., third-party fault), information on alcohol and drug influence, treatment specifics; and data regarding the type of fireworks involved and their handling. The weather data was assessed and provided by the DWD (German Weather Station in Leipzig-Holzhausen). We selected two key parameters: (1) daily minimum of the air temperature at 2 m altitude in [°C] and (2) form of precipitation. The precipitation data was differentiated according to the following categories: 0 equals no precipitation, 1 only rain, 4 form not known, although precipitation reported, 6 only rain, 7 only snow, 8 rain and snow, 9 missing value or precipitation form not detectable with automatic measurement. The classification of fireworks was based on the system used by the German Federal Institute for Materials Research and Testing, which is also employed by the German custom authorities and includes the following distinctions/categories: Category F1: Indoor fireworks, which may be used throughout the year by individuals over the age of 12. Category F2: consumer fireworks that may only be set off on 31 December and 1 January. Category F3 and F4: display and professional fireworks that may only be used by trained firework operators [10].

### Statistical analysis

The statistical analysis was conducted using SPSS 29.0 (SPSS Inc., Chicago, IL, USA). Unless otherwise denoted, data was presented as mean with standard deviation (SD) or frequencies (n) with percentage (%). It is noted that the percentage of missing data (to reach 100%) is due to the absence of information in the medical records for some parameters. Nominal variables were associated using Chi-square tests, while non-parametric tests (Mann-Whitney U and Kruskal-Wallis H test as chosen by SPSS) were used to compare continuous data.

To determine the prognostic value of the identified risk factors, odds ratios (OR) with 95% confidence intervals (CI) were calculated. Figures were created using an open-source online tool (http://BioRender.com).

## Results

A total of 155 patients with firework-related injuries (mean age 24 years, range 2 to 74 years, 80.0% male) met the inclusion criteria and were subjected to further analysis.

**Fig. 1:**
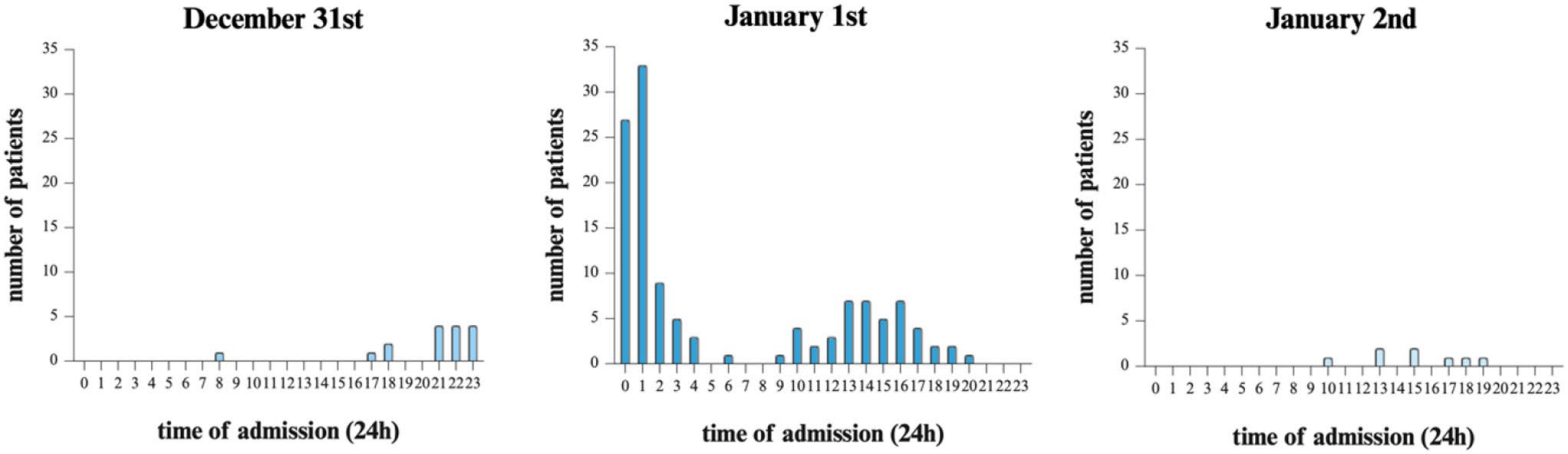
Hourly distribution of patient admissions from Dec 31st to Jan 2nd over 10 years (There were no patient admissions on Dec 28th and 29th, only 1 admission on Dec 30th, and only 8 admissions on Jan 3rd )

More than one-third of these patients (38.7%) were admitted within the two hours after midnight, while the remainder was distributed throughout the rest of the day (Fig. 1).

The mean temperature at the time of injury was 3 ± 5 °C (range, -7 to 13 °C), with precipitation recorded in 100 out of 155 cases (64.5%).

### Mechanism of firework injury

**Fig. 2:**
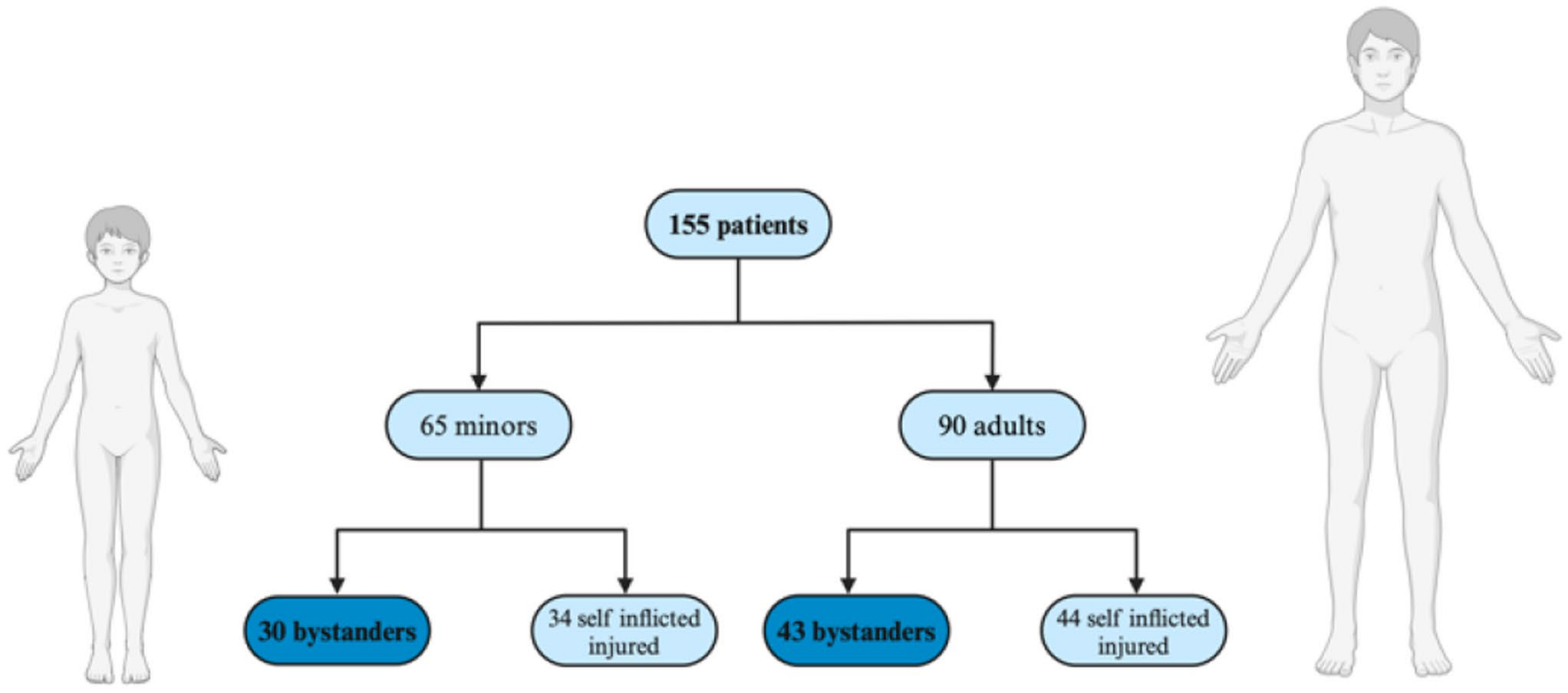
Distribution of patients and proportion of minors among firework-related injuries (excluding 4 patients with uncertain information)

Self-inflicted injury (e.g. delayed disposal after ignition) was the most common mechanism (48.4%), followed by accidental injuries of unknown or uncertain origin (31.6%). Injuries sustained as a bystander struck by a firework, where another individual was clearly implicated, accounted for 16.8% of cases. Additionally, there was one case where another person forcibly held the patient’s hand around the firework. Almost half of the patients (47.1%) were injured as bystanders, with 30 of the 65 injured minors also classified as bystanders (Fig. 2; information uncertain in 4/155 patients). Only 4 patients (2.6%) sustained injuries from indoor fireworks (category F1), while the remainder were injured from a firecracker (102/155, 65.8%), rockets (34/155, 21.9%), firework batteries (9/155, 5.8%) – all category F2 - or other kinds of fireworks including flare guns, black powder bombs, and unknown objects (6/155, 3.9%). As far as this could be retrospectively assessed, the majority (122/155, 78.7%) of the purchased fireworks were in compliance with German legislation. However, considering who used the fireworks (e.g., minors) and the methods of handling, this study concludes that none of the cases represented legal use of fireworks. The remaining 33 patients’ documentation only provided information on the injury mechanism; thus, the used fireworks could not be properly assessed.

Age restrictions were violated in 36.4% of the cases, and adherence to legally mandated safety distance was absent in all cases.

Data on alcohol and drugs were only available for 13 out of 155 patients, all of whom had consumed alcohol. As a result, further analysis could not be conducted.

### Injury patterns

**Fig. 3:**
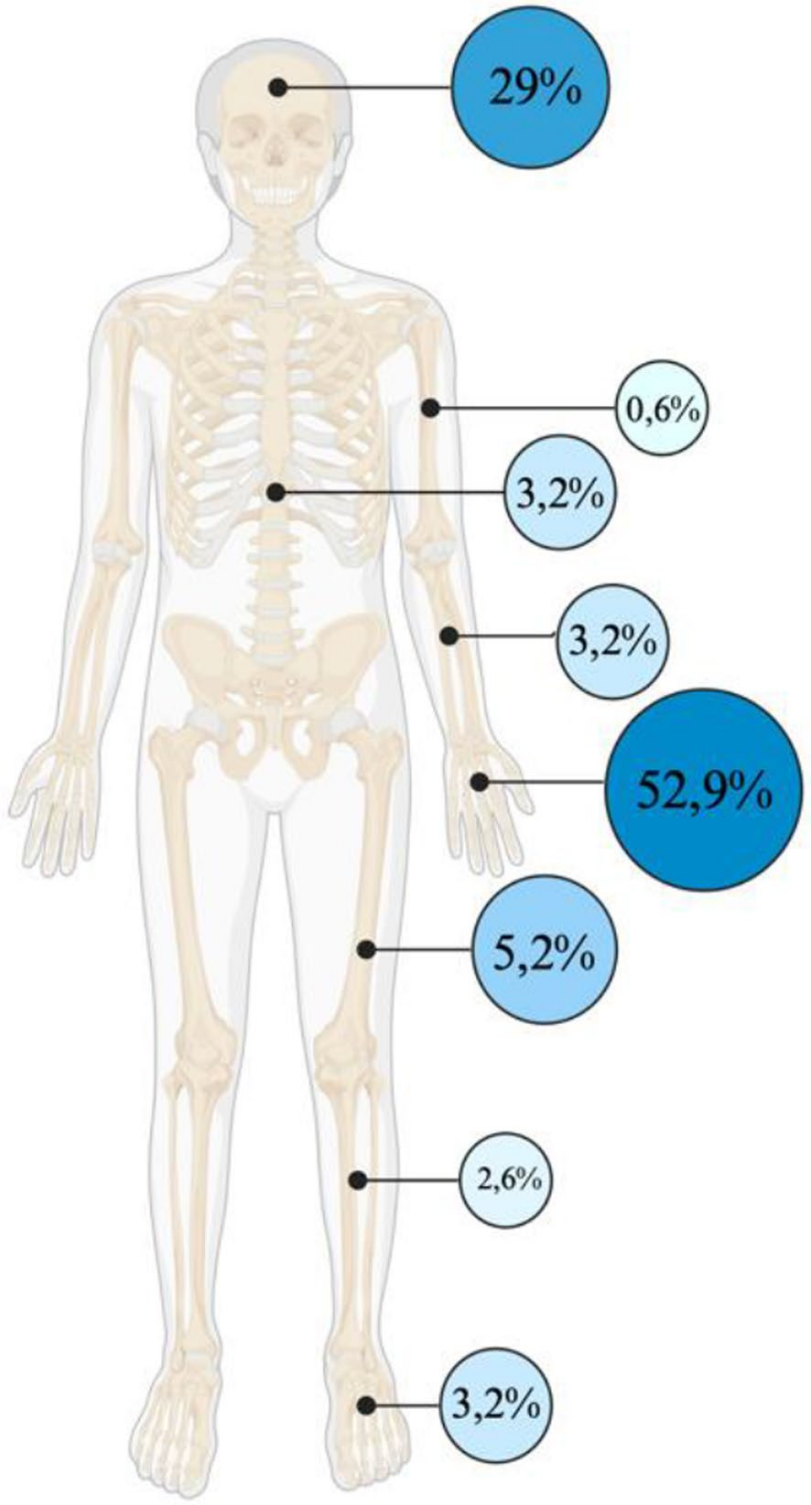
Anatomical distribution of injuries

Injuries to the hand were by far the most frequent, accounting for 52.9% with 66 out of 155 injuries cases (42.6%) involving the fingers. A total of 79 patients (51.0%) sustained injuries across multiple body regions. 29% (45/155 patients) presented with injuries to the head (Fig. 3), of those, 42,2% (19/45 patients) had burn injuries, 8,4% had eye, and 0,6% had ear injuries. All patients exhibited firework-related soft tissue injuries, with bone injuries occurring in 26 out of 155 cases (16.8%). Burn injuries were observed in 68 cases (43.9%), with 58.3% classified as first-degree burns, 36.1% as second-degree, and 5.5% as third-degree. The likelihood of burn injuries was significantly reduced in the presence of precipitation (OR 0.4, 95% CI 0.2 to 0.7, *p* = 0.004).

The visual representation (Fig. 3) is based on the international AO classification (Working Group for Osteosynthesis Issues [20]), and gives a percentage overview of fractures and soft tissue/burn related injuries: AO 1 includes upper arm, scapula and clavicle regions; AO 2 including forearm and wrist, AO 3 encompasses femur, knee and upper leg; AO 4 covers lower leg including malleolus, tibia and fibula; AO 5 represents core and spine; AO 6 pelvic region, where no injuries were observed; AO 7 hand, metacarpals and phalanges; AO 8 relates to the foot, metatarsals and phalanges; AO 9 including injuries of the head, eye, neck, regio oralis, ear, lips and cheeks. The discrepancy of 0,06% can be explained by rounding errors or missing data.

### Treatment and outcome

Most patients (115/155, 74.2%) were admitted to the ward, while an additional five required ICU monitoring. The average hospital stay lasted 2 ± 5 days (range, 1 to 33).

Surgical intervention (such as fracture fixation, burn injury related debridement, reconstructive surgery) were necessary in 37 of the admitted cases (23.9%). Risk factors (Table 1) associated with injuries requiring surgery included: bone injuries (OR 107.1, 95% CI 22.7 to 505.6, *p* < 0.001), male sex (OR 5.7, 95% CI 1. to 25.0, *p* = 0.009), and injuries involving multiple body regions (OR 4.9, 95% CI 2.1 to 11.7, *p* < 0.001).

Upon discharge, 72 out of 155 patients (46.5%) had some degree of permanent loss of function. Furthermore, patients who had required surgical intervention were more likely to suffer from permanent functional impairments (OR 16.7, 95% CI 5.5 to 50.5, *p* < 0.001). Overall, according to the used AIS classification [19], 75% of all patients (117/155, AIS 1) sustained minor injuries. 17 out of 155 patients were classified as AIS 2 (moderate injuries), 18/155 (12%) as AIS 3, indicating serious but non-life-threatening injuries. Two patients survived severe, life-threatening injuries (AIS 4). One patient deceased due to the firework-related injuries to the skull, caused by a self-constructed and wrongfully triggered ignition device (AIS 6).

**Table 1:** Differences between female and male patients

	Female	Male
N	31	124
Age [years]	27 ± 16,77	24 ± 14,01
AIS	1,1 ± 0,49	1,5 ± 0,88
Bone fractures	2 (6,5%)	25 (20,2%)
Multiple injuries	10 (32,3%)	69 (55,6%)

(In the case of patients sustaining multiple injuries, the AIS of the most severe injury was used for analysis only)

## Discussion

The aim of the study was to investigate the epidemiology of firework-related injuries, identifying common injury patterns, and pinpointing key risk factors.

Notably, 80% of patients who sustained injuries were male, with the mean age of 24 years. This is in line with previous reports in the literature, which identified male gender as significant risk factor for firework-related injuries [6, 11, 12, 14, 21–24] and demonstrated a correlation between younger age and a higher risk [11, 14]. Injuries to the hand were by far the most frequent with about half of the patients sustaining injuries to multiple body regions, a pattern consistent with findings from previous studies [15]. The hand injury pattern found in the present study is in line with a multicenter study conducted over a seven-year period in five major German hospitals [14]. In addition to our findings, studies focused on ophthalmology and otolaryngology patients have demonstrated that eyes and ears are more frequently affected [6, 12, 15, 22, 24].

The predominant cause underlying these injuries was self-inflicted, specifically igniting the firework and throwing it away prematurely. This observation is consistent with the results of previous studies on the topic [21, 24]. Strikingly, our data study shows that nearly half of the patients sustained injuries as bystanders, a finding that is corroborated by previous research as well [1, 6, 12, 21–24].

Previous studies investigating the timing of firework-related injuries support our findings: a multicenter study reported, more than one-third (38.7%) of the patients sustained their injuries within the two hours after midnight on January 1st, with the remaining injuries taking place later in the afternoon on New Year’s Day. In a study conducted by Demmer et al., the median time of injury for adults was at 01:50 am on New Year’s Eve [14].

Crucially, our data highlights the following significant risk factors for surgical intervention: bone injuries, male sex, and injuries to multiple body regions. While the necessity for surgical intervention is clearly linked to multiple injuries and bone fractures the association with male sex is less well understood. However, it is known from other behavioral studies, that males are more prone to risk-taking behaviors [25]. Interestingly, a significant negative correlation was identified between the likelihood of burn injuries and meteorological conditions involving precipitation. According to existing literature, no comparable analysis or data are available.

Previous studies have demonstrated a correlation between substance use (e.g., alcohol and drugs) and elevated risk of firework-related injuries, particularly among younger individuals and during celebratory occasions such as New Year’s [1, 6, 7, 11, 22, 24, 26]. Surprisingly, our data provided unsuccessful information demonstrating whether alcohol and drugs have a significant influence on the frequency of injuries sustained around New Year’s Eve. Given that in a worldwide comparison the European Union has the highest per capita alcohol consumption, as compared to other regions of the world, and Germany ranks top 3 among the EU countries [27, 28], the potential relevance of this behavioral aspect and socioeconomical status associated with potential injuries would be an intriguing question to incorporate into future studies.

There are some limitations to our study. First, only pediatric and adult patients who were seen by the surgeon on-call in the pediatric and adult surgical emergency departments of the University Hospital Leipzig were included, excluding patients from departments such as otorhinolaryngology and ophthalmology. In addition, outpatient cases, as well as patients not directly injured by fireworks (e.g. patients who “stumbled over a rocket”), were also excluded, possibly underestimating the total number of New Year’s Eve-related injuries and the higher associated emergency department workload.

Additionally, anamnesis data varied considerably due to the involvement of multiple on-call physicians contributing to the SAP documentation system, leading to inconsistencies and incomplete records. The assessment of firework related injuries is not standardized in the authors’ institution. Consequently, we were compelled to rely on descriptions such as “rockets” or “firecrackers” documented in the patient’s history to identify and categorise the fireworks in question in accordance with German legislation (firework categories F1-F4).

As a result, the study’s limitations include its retrospective design and the existence of missing or incomplete data. The inability to follow-up patients’ cases and their outcome when they are transferred to other treating physicians and practices further limits the data.

The clinical relevance of our topic is undeniable: firework-related injuries are entirely preventable, and their prevention could significantly reduce the workload on emergency departments. Future studies should explore the extent of the increased workload during the festive period surrounding New Year’s Eve and assess whether stricter regulations on the private purchase and use of fireworks could help to reduce health care costs demand.

**Fig. 4:**
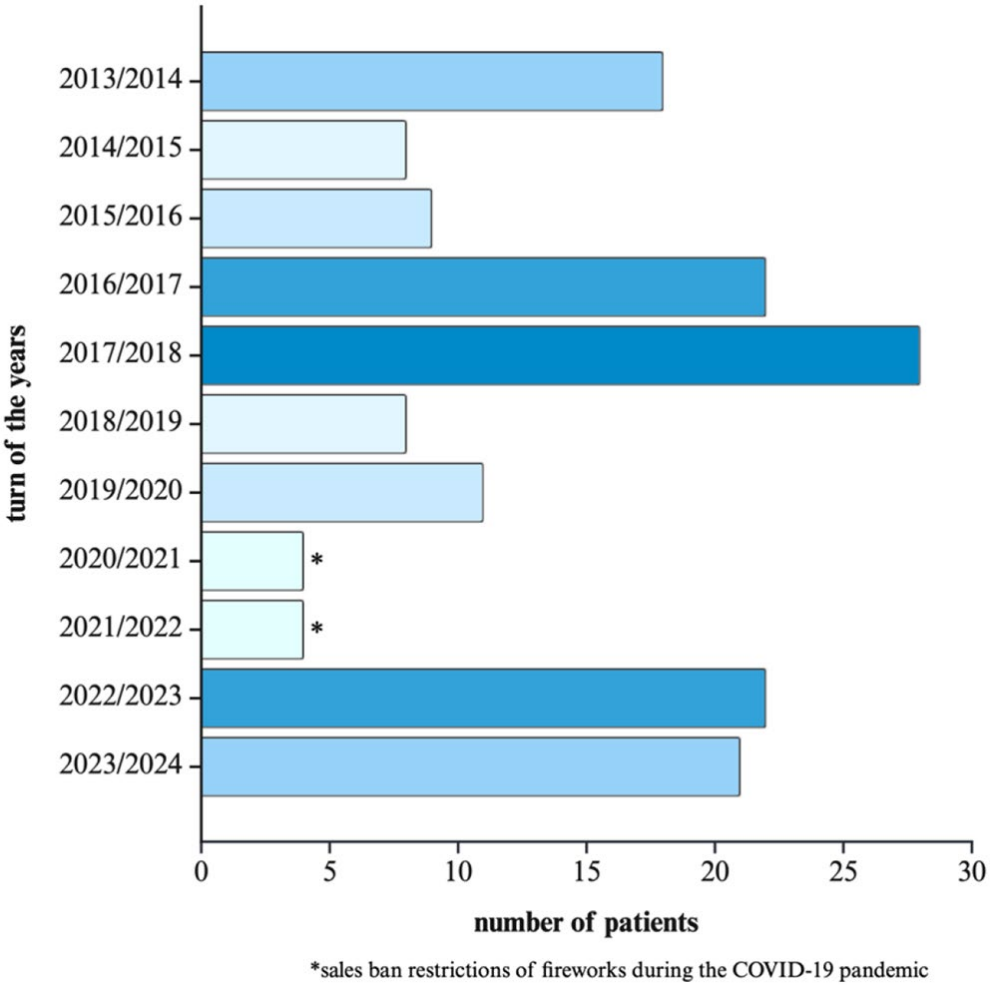
Distribution of patients across New Year transitions

Evidence from successful prevention campaigns, such as in Denmark [29], or restrictive firework legislation (e.g. the sales ban during the COVID-19 pandemic), have shown a significant reduction in the number of firework related injuries [12, 14]. Figure 4 pinpoints our observation of a reduction in the number of patients with firework-related injuries around New Year’s, which lends support to the hypothesis that legal restrictions and sales bans (e.g. during the COVID-19 pandemic) result in a decline in such injuries. Launching additional prevention campaigns, led by organizations such as DGUV (Deutsche Gesetzliche Unfallversicherung – German Social Accidental Insurance) could raise the overall awareness of existing campaigns [30] with the aim of reducing firework-related injuries and their consequences. This raises the question which types of campaigns (e.g. visual media, social media, schools) are most effective in targeting the high-risk group of young males.

Another interesting field for future research is the psychological and emotional impacts of severe firework injuries, especially on children and adolescents. The results may help to assess the potential for additional legislative measures and guide future political action, such as restricting and reducing public sales of private fireworks and pyrotechnics in Germany, to decrease the prevalence of firework-related injuries and fatalities among patients and bystanders, as well as associated hospitalizations, healthcare costs, and psychological trauma.

## Conclusion

Firework-related injuries during New Year’s Eve are linked to significant morbidity with a notable impact on the hands and a frequent need for surgical intervention. Bone injuries, male sex and multiple body region injuries are associated with higher need for surgical treatment. Although most fireworks are purchased legally, there is widespread noncompliance with age restrictions and safety protocols. These findings highlight the necessity of public health initiatives, aimed at reducing the incidence and severity of such injuries, rather than relying solely on stricter regulations. Moreover, the efficacy of prevention efforts can be strengthened through the implementation of a comprehensive approach, as exemplified in Denmark [29], which for example includes: (1) The introduction of annual prophylaxis campaign materials into schools and groups in danger, such as boys and young men, (2) the implementation of mandatory public awareness campaigns focusing on the dangers of fireworks and the use of protective gear, such as eye glasses, and (3) encouraging the use of community-based alternatives to private fireworks displays, such as safe, organized public celebrations.

## Data Availability

The climate data (Holzhausen, Leipzig Germany, number 02928) used in this study is accessible at https://opendata.dwd.de/climate_environment/CDC/observations_germany/climate/daily/kl/.
